# From preclinical development to clinical application: Kit formulation for radiolabelling the minigastrin analogue CP04 with In-111 for a first-in-human clinical trial

**DOI:** 10.1016/j.ejps.2016.01.023

**Published:** 2016-01-27

**Authors:** Dariusz Pawlak, Christine Rangger, Petra Kolenc Peitl, Piotr Garnuszek, Michał Maurin, Laura Ihli, Marko Kroselj, Theodosia Maina, Helmut Maecke, Paola Erba, Leopold Kremser, Alicja Hubalewska-Dydejczyk, Renata Mikołajczak, Clemens Decristoforo

**Affiliations:** a Radioisotope Centre POLATOM, National Centre for Nuclear Research, Otwock, Poland; b Dept. of Nuclear Medicine, Medical University Innsbruck, Austria; c Dept. of Nuclear Medicine, University Medical Centre Ljubljana, Slovenia; d Molecular Radiopharmacy, INRASTES, NCSR “Demokritos”, Athens, Greece; e Dept. of Nuclear Medicine, University Hospital Freiburg, Germany; f Dept. of Nuclear Medicine, Azienda Ospedaliero-Universitaria Pisana, Pisa, Italy; g Division of Clinical Biochemistry, Biocenter, Medical University Innsbruck, Austria; h Dept. of Endocrinology, Jagiellonian University Medical College, Krakow, Poland

**Keywords:** Radiopharmaceutical Kit, Formulation, In-111, Freeze-Drying, Minigastrin, Medullary thyroid carcinoma

## Abstract

**Introduction:**

A variety of radiolabelled minigastrin analogues targeting the cholecystokinin 2 (CCK2) receptor were developed and compared in a concerted preclinical testing to select the most promising radiotracer for diagnosis and treatment of medullary thyroid carcinoma (MTC). DOTA–DGlu–DGlu–DGlu–DGlu–DGlu–DGlu– Ala–Tyr–Gly–Trp–Met–Asp–Phe–NH_2_ (CP04) after labelling with ^111^In displayed excellent characteristics, such as high stability, receptor affinity, specific and persistent tumour uptake and low kidney retention in animal models. Therefore, it was selected for further clinical evaluation within the ERA-NET project GRAN-T-MTC. Here we report on the development of a pharmaceutical freeze-dried formulation of the precursor CP04 for a first multi-centre clinical trial with ^111^In-CP04 in MTC patients.

**Materials and methods:**

The kit formulation was optimised by adjustment of buffer, additives and radiolabelling conditions. Three clinical grade batches of a final kit formulation with two different amounts of peptide (10 or 50 μg) were prepared and radiolabelled with ^111^In. Quality control and stability assays of both the kits and the resulting radiolabelled compound were performed by HPLC analysis.

**Results:**

Use of ascorbic acid buffer (pH 4.5) allowed freeze-drying of the kit formulation with satisfactory pellet-formation. Addition of methionine and gentisic acid as well as careful selection of radiolabelling temperature was required to avoid extensive oxidation of the Met^11^-residue. Trace metal contamination, in particular Zn, was found to be a major challenge during the pharmaceutical filling process in particular for the 10 μg formulation. The final formulations contained 10 or 50 μg CP04, 25 mg ascorbic acid, 0.5 mg gentisic acid and 5 mg l-methionine. The radiolabelling performed by incubation of 200–250 MBq ^111^InCl_3_ at 90 °C for 15 min resulted in reproducible radiochemical purity (RCP) >94%. Kit-stability was proven for >6 months at +5 °C and at +25 °C. The radiolabelled product was stable for >4 h at +25 °C.

**Conclusion:**

A kit formulation to prepare ^111^In-CP04 for clinical application was developed, showing high stability of the kit as well as high RCP of the final product.

## 1. Introduction

Medullary thyroid carcinoma (MTC) is still one of the most challenging cancers for both physicians and patients (Hazard et al., 1959; Miccoli et al., 2007; Sippel et al., 2008; Machens et al., 2007). Epidemiological studies have shown that during the past 30 years neither a change in stage at diagnosis nor improvement in survival has occurred for MTC patients (Roman et al., 2006; Moley et al., 1999; Rufini et al., 2000). One third of patients present with locally invasive tumours or clinically apparent spread to the regional lymph nodes and 13% with distant metastatic spread. In particular patients with metastatic MTC are left with few ineffective therapeutic options. Chemotherapy has been of limited value, and radiation therapy may be used only to control local disease. Therefore, it is necessary to develop alternative therapeutic strategies to control tumour growth, possibly through manipulation of various cellular signalling pathways and the use of new biomarkers.

**Fig. 1:**
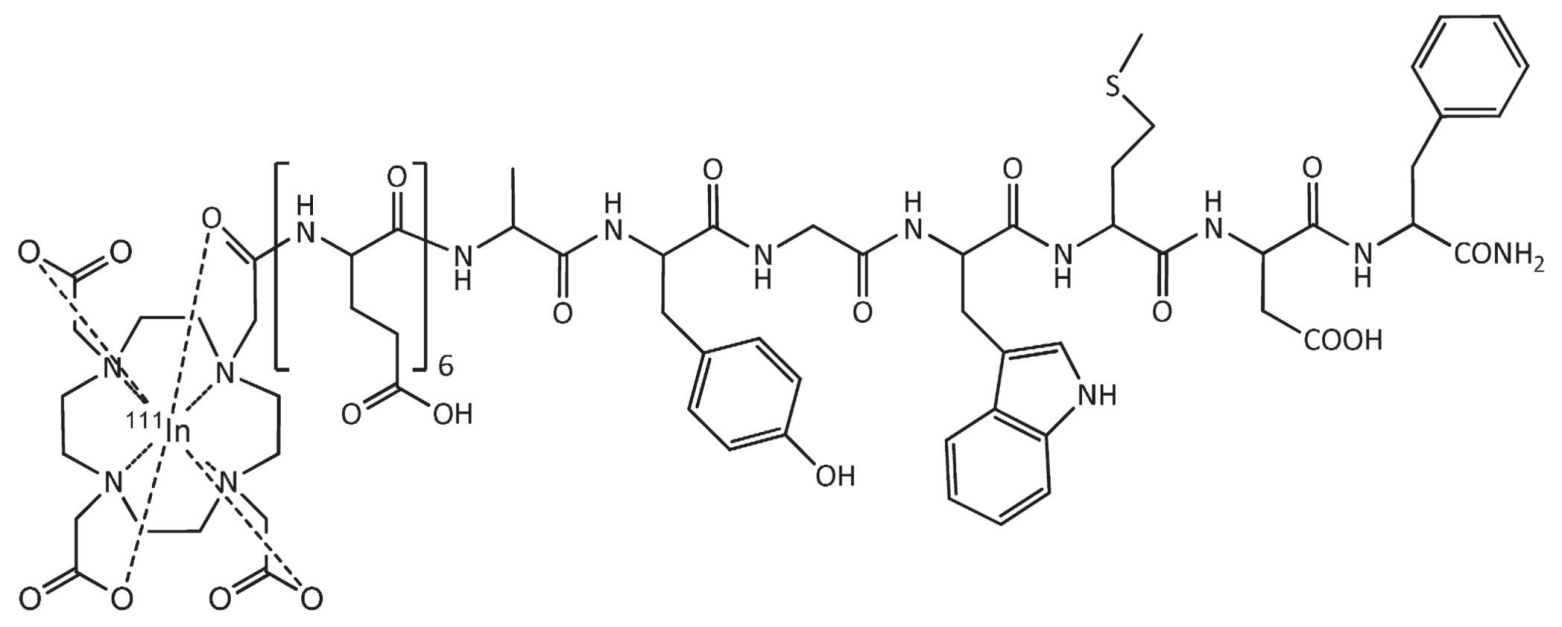
Structure of ^111^In-labelled DOTA–DGlu–DGlu–DGlu–DGlu–DGlu–DGlu–Ala–Tyr–Gly–Trp–Met–Asp–Phe–NH_2_ (CP04), sum formula: C_89_H_118_N_19_O_35_S^111^ In, MW (av) 2157.1 g/mol.

The cholecystokinin 2 (CCK2) receptor is overexpressed in MTC with high density and incidence of over 90%, as revealed by autoradiographic studies (Reubi and Waser, 2003; Béhé and Behr, 2002). From the late 1990’s, a variety of CCK2/gastrin related peptides (members of the gastrin and cholecystokinin families, or possessing such characteristics) were studied in vitro and in preclinical animal models. First generation of radiolabelled minigastrins, based on human minigastrin (Leu–Glu– Glu–Glu–Glu–Glu–Ala–Tyr–Gly–Trp–Met–Asp–Phe–NH_2_) were associated with very high uptake and retention in the kidneys (von Guggenberg et al., 2004). Truncation of the sequence by removal of amino terminal Glu-residues resulted in lower kidney uptake, but metabolic stability was severely compromised (Good et al., 2008). In a multi-centre preclinical study within a coordinated research project (COST BM0607) a number of different minigastrin analogues conjugated with 1,4,7,10-tetraazacyclododecane-N,N′,N″,N‴-tetraacetic acid (DOTA) and suitable for radiolabelling with ^111^In, ^68^Ga (for diagnostic imaging), or with ^90^Y and ^177^Lu (for radionuclide therapy) were compared with respect to stability (Ocak et al., 2011), receptor binding, internalisation (Aloj et al., 2011), in vivo tumour targeting and pharmacokinetics in animal models (Laverman et al., 2011). From this comparison ^111^In-DOTA–DGlu–DGlu–DGlu–DGlu–DGlu–DGlu–Ala–Tyr–Gly–Trp–Met–Asp–Phe–NH_2_ (^111^In-CP04) showed the most promising characteristics for clinical translation, such as high metabolic stability, receptor affinity, high and prolonged tumour uptake and low kidney retention (Kolenc Peitl et al., 2015) (see Fig. 1). As a result, ^111^In-CP04 was selected for further clinical evaluation in MTC-patients, currently being implemented within the frameworks of a multinational European cooperation project on personalised medicine (TRANSCAN call of the EU within ERA-NET, project GRAN-T-MTC). The aim of the project is to evaluate the safety and tumour-targeting efficacy of ^111^In-CP04 in patients with metastatic MTC. For the first-in-human study two dosage forms were required: a lower dose containing 10 μg CP04 and a higher dose of 50 μg CP04, both to be radiolabelled with 200–250 MBq ^111^In for patient application. The 10 μg dose is intended to be used in first applications of ^111^In-CP04. Only if safety is ensured the 50 μg dose will be applied, an amount of precursor also suitable for later translation towards radionuclide therapy by radiolabelling with e.g. ^177^Lu.

For conducting a clinical trial in Europe, a pharmaceutical dossier, named Investigational Medicinal Product Dossier (IMPD, based on the common technical dossier (CTD) format) has to be submitted, containing extensive chemical and pharmaceutical data on the compound concerned as well as information of pre-clinical pharmacology, pharmacokinetics and toxicology in animal models. For radiopharmaceuticals special information related to radioactivity and radiolabelling properties has to be provided (Todde et al., 2014). Herein we report on the chemical and pharmaceutical development and characterisation of a freeze-dried kit formulation for radiolabelling of the radiopharmaceutical precursor CP04 with ^111^In (chapter 2.1 of the CTD). Data on preclinical pharmacology, pharmacokinetics and toxicology (chapter 2.2. of the CTD) will be reported separately.

## 2. Material and methods

### 2.1. Chemicals and materials

If not otherwise stated, chemicals, materials and solvents were of pharmaceutical grade for kit preparation and reagent grade for other experiments and were used without further purification.

^111^InCl_3_ solution was supplied by Mallinckrodt Pharmaceuticals (Petten, NL) as a sterile, non-pyrogenic solution of non-carrier-added ^111^InCl_3_ in 0.05 M HCl. Each 0.5 mL of the solution contained 185 MBq (5 mCi) of ^111^InCl_3_ at time of calibration (specific activity of >1.85 GBq/μg indium at time of calibration).

l-Methionine was provided by SAFC chemicals (Cleveland, OH) in pharmaceutical grade, ascorbic acid (pharmaceutical grade) and gentisic acid (Ultrapure quality) were provided by Sigma Aldrich (St. Louis, MO).

Sterilised distilled water for injection (Pharm.Eur.) was provided by Fresenius Kabi (Halden, Norway) in 10 mL plastic ampoules.

For freeze-drying, 2- or 10-mL clear borosilicate glass vials from SCHOTT (StandardLine, FIOLAX®, Müllheim, Germany) corresponding to Ph.Eur. Type I, with chlorobutyl rubber stoppers were used.

### 2.2. CP04-precursor

CP04 was synthesised by piCHEM (Graz, Austria) in GMP grade. Briefly, Fmoc solid phase peptide synthesis (SPPS) was applied by the use of a batch-mode synthesiser. Cleavage from the resin and deprotection of side chain functionalities were performed in one step using trifluoroacetic acid (TFA), followed by preparative RP-HPLC purification. Tri-t-Bu-DOTA was coupled in solution to the peptide, deprotected by TFA and again HPLC purified, followed by freeze-drying and filling into Sterile Square Media Bottles (PETG). Analytical testing included mass spectrometry, amino acid analysis, purity, content of peptide, water, TFA and residual solvents and endotoxins.

### 2.3. Analytical methods

#### 2.3.1. HPLC-analysis

HPLC-system 1 (CP04 quantification and kit quality control): LC-10 AD pumps (Shimadzu), with a variable wavelength detector SPD-10A, oven CTO 10AS (Shimadzu), manual injector Rheodyne 1725 with a 20 μL loop, column: Kinetex C18, 5 μm, 150 mm × 4.6 mm (PhenomenexTorrance, CA); flow rates of 1 mL/min; temperature: 40 °C, UV detection at 220 nm; isocratic elution (25% of 0.1% TFA (explained already above) in acetonitrile (ACN) and 75% of 0.1% aq.TFA). Quantification of CP04 in kits was achieved with the aid of a standard curve using CP04 CRS standard with peptide content established by elemental analysis, whereby specificity, linearity, range, accuracy, precision, limit of detection (LOD) and quantitation (LOQ) were determined.

HPLC-system 2 (impurity characterisation and radiochemical purity determination): UltiMate 3000 autosampler with an UltiMate 3000 variable wavelength detector (Dionex, Thermo Scientific, Germering, Germany) and RayTest GABI radioactivity detector (Raytest, Straubenhard, Germany), column: Kinetex C18, 5 μm, 150 mm × 4.6 mm (Phenomenex); flow rates of 1 mL/min; UV detection at 280 nm; isocratic elution; solvents: 25% of 0.1% TFA in ACN and 75% of 0.1% aq. TFA.

Endotoxin testing was performed by Gel Clot technique using a *E. coli* 055:B6 endotoxin standard control (Charles River, Charleston, SC).

Sterility testing was performed according to the European Pharmacopoeia using the direct inoculation technique.

### 2.4. Nano-HPLC–ESI-MS analysis

For determination of CP04 impurities in the kit formulations nano-HPLC electrospray ionisation mass spectrometry (ESI-MS) was carried out using an UltiMate 3000 nano-HPLC system coupled to a LTQ Orbitrap XL mass spectrometer (both Thermo Scientific) equipped with a nanospray ionisation source. The analytes were separated on a homemade fritless fused-silica microcapillary column (75 μm i.d. × 280 μm o.d. × 10 cm length) packed with 3 μm reversed-phase C18 material (Reprosil). Solvents for HPLC were 0.1% formic acid (solvent A) and 0.1% formic acid in 85% ACN (solvent B). The gradient profile was as follows: 0–2 min, 4% B; 2–40 min, 4–40% B; 40–45 min, 40–100% B, and 45–55 min, 100% B. The flow rate was 250 nL/min. The mass spectrometer was operated in the data dependent mode selecting the top 4 most abundant isotope patterns with charge 2+, 3+, and 4+ from the survey scan with an isolation window of 2 mass-to-charge ratio (m/z). Survey full scan MS spectra were acquired form 300 to 2000 m/z at a resolution of 60,000.

To characterise the chromatographic behaviour indium, copper (II), zinc and iron (II) complexes of CP04 were prepared. For this purpose, 50 μg CP04 were incubated with 10-fold molar excess of the respective chloride salt in ascorbic acid buffer (pH 4.5) followed by brief incubation at 90 °C for 10 min and subsequently analysed by HPLC system 1.

### 2.5. Wet-radiolabelling/preformulation studies

Freshly prepared stock solutions of the CP04 peptide were prepared (100 μg CP04 in 100 μL PBS). 10–20 μL CP04 stock solution and [^111^In]-chloride (60 μL, 40–50 MBq in 0.02 M HCl, pH 1) was buffered by adding either 100 μL of ascorbic acid buffer (50 mg buffer in 0.2 mL H_2_O, pH 3.5) or 100 μL of sodium acetate buffer (0.8 M, pH 4.5). The incubation process requires slightly acidic conditions for optimal radionuclide binding to the DOTA-conjugated peptide. In order to minimise the Met^11^ oxidation during radiolabelling, seleno-dl-methionine (10 μL 0.01 mg/μL) was added to the samples. To determine the optimal reaction conditions, the labelling mixture was incubated between 15 and 30 min at 75/85/95 °C (see Table 1). After incubation, 80 μL of H_2_O and 80 μL 0.01 M ethylenediaminetetraacetic acid (EDTA) solution were added to 20 μL of the radiolabelled mixture. To determine radiochemical yield (RCY) and radiochemical purity (RCP) of the radiolabelled product and to monitor the presence of oxidised peptide, RP-HPLC was carried out (HPLC system 1).

### 2.6. Kit preparation

19 different development batches (batch no. 1–8) of CP04 kits were prepared varying different parameters including: CP04 content (10, 20, 50 μg), ascorbic acid content, additives (l-methionine, gentisic acid), vial size, as summarised in Table 2. After development, 4 batches (batch no. 9–10) were prepared under GMP conditions meeting requirements for use in humans and tested extensively regarding all defined specifications.

The bulk solution of the formulation was prepared in a clean room using pre-sterilised glass vials, containers and stoppers. To a 250 mL plastic bottle 2.5 g of ascorbic acid was added, dissolved in 94 mL of water for injection. The pH of the bulk solution was adjusted to 4.5 with 1 mL of 30% NaOH. Next, 53 mg of gentisic acid and 520 mg of l-methionine were added and the solution was purged with nitrogen for 20 min. CP04 (1 mg or 5 mg) was dissolved in 5 mL of 0.1% NaOH and added to the bulk solution. The final bulk solution was then purged with nitrogen for 5 min and filtered through a 0.2 μm Sterifix Paed filter. The bulk solution was dispensed into the 2 mL or 10 mL vials in the range of 1 mL ± 1% per vial and the vials were capped with the stoppers and allowed to freeze for 20 h at −70 °C in the freeze-dryer. Afterwards the product was lyophilised according to the following lyophilisation scheme: initial shelf temperature was set at −50 °C. During the first phase of lyophilisation a stable temperature of −38 °C and a pressure of 0.1 mbar were maintained. Further steps relied on temperature change from −35 °C to −25 °C at 2.5 °C/h, then the temperature was changed from −25 °C to 40 °C at 5.0 °C/h. During the second phase of lyophilisation the product was dried at 40 °C for 8 h. All steps were conducted at 0.1 mbar pressure. Finally, the freeze-dryer chamber was filled with sterile and dry nitrogen and, using the mechanic system of the freeze-dryer, the vials were sealed with rubber stoppers, taken out of the freeze-dryer and capped with metal caps. The vials were inspected for container closure defects and product appearance. Samples were sent to quality control for product release testing.

Quality control of the kit included appearance of kit, water content by Karl-Fischer coulometric oven method (Metrohm) and appearance after dissolution in water (1 mL, clarity according to Ph.Eur. 2.2.1., colouration according to Ph.Eur. 2.2.2), identification, and quantification of CP04 by RP-HPLC analysis and detection at 220 nm, measurement of pH, sterility testing, detection of endotoxins and assessment of RCP after kit reconstitution and radiolabelling with ^111^In using RP-HPLC analysis and radiometric detection.

### 2.7. Kit radiolabelling

Radiolabelling of CP04 with ^111^In was carried out in a final volume of 1 mL. The suitable volume of water was calculated depending on the radioactivity concentration of the ^111^In-solution from the provider. Typically 0.6–0.8 mL of water together with 0.2–0.4 mL of ^111^InCl_3_ (200–250 MBq) were rapidly added to the vial. The labelling mixture was then incubated for 10–30 min at 75–95 °C.

The radiolabelled kit solution was analysed by RP-HPLC. Samples for analysis were prepared by adding about 20 μL of labelling solution to 100 μL of 0.1 mg/mL DTPA Na_3_Ca·H_2_O in water, serving as a free ^111^In scavenger to prevent sticking of radioactivity on the column.

### 2.8. Stability studies

The final formulation of both dosages (10 μg: batches 5C, 8B, 9B; 50 μg: batches 8A, 9A) underwent extensive stability testing during storage of the CP04 kits in a refrigerator (5 °C) in the primary container tightly closed with rubber stopper and aluminium seal up to 1 year. In addition accelerated stability testing at 25 °C for 6 months (batches 8A, 8B, 9A and 9B) was performed. Analytical tests included appearance before and after dissolution, measurement of pH, radiolabelling with ^111^In and subsequent HPLC analysis for confirmation of identity and RCP of ^111^In-CP04.

Stability of the radiolabelled product was tested for the same kit batches reported above by determination of RCP by HPLC at different time points (0, 2, 4 and 24 h) after radiolabelling.

## 3. Results

### 3.1. Wet radiolabelling/preformulation studies

Radiolabelling of the precursor CP04 could be performed at high specific activities (>50 MBq ^111^In/20 μg CP04) and overall with high radiochemical yield. An incubation temperature of 75 °C led to RCP for ^111^In-labelled CP04 of >90% in all buffer systems. A prolongation of the incubation time generally resulted in higher oxidation of Met^11^ in CP04. Oxidation of the peptide could be reduced to <1.5% by addition of seleno-dl-methionine in the acetate buffer formulation. However, this composition did not achieve significantly higher RCP as compared to the ascorbic acid formulation. Therefore, the ascorbic acid formulation was selected for further kit development.

### 3.2. Characterisation of radiochemical impurities

Characterisation of impurities was achieved by HPLC and radiometric detection (HPLC system 1) with the aid of suitable radioactive control samples, such as ^111^InCl_3_, ^111^In-DTPA and ^111^In-CP04 artificially oxidised by treatment with a diluted H_2_O_2_-solution. The respective retention times (t_R_) were: “free” ^111^In (^111^In-chloride solution and ^111^In-DTPA): t_R_ 1.4 min, oxidised ^111^In-CP04: t_R_ 3.1 min (with 2 visible peaks corresponding to the sulfoxide and the sulfone of the Met^11^-residue), ^111^In-CP04: t_R_ 9.0 min. The identity of oxidised species was additionally confirmed by identification of the unlabelled oxidised CP04 at t_R_ 3.1 min by mass spectrometry.

Additional small peaks were detected eluting closely to peaks visible in the UV trace of the CP04 precursor solutions, thus indicating impurities from CP04 synthesis. In the final batches of CP04 these radioactive peaks were always <1% and therefore were not further characterised.

**Fig. 2:**
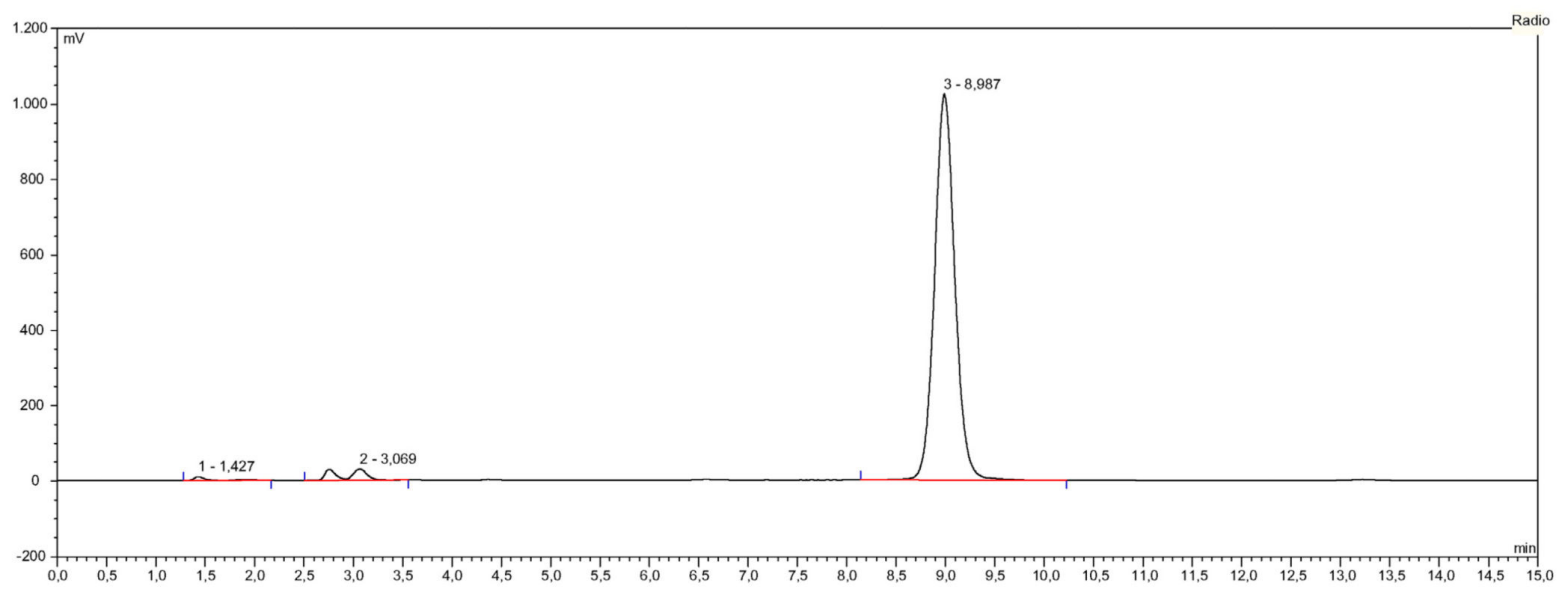
HPLC chromatogram of ^111^In-CP04: sample radiolabelling of kit #9A (system 1) radioactivity detection), t_R_ 1.4 min: free ^111^In and ^111^In-DTPA, t_R_3.1 min: two oxidised forms of ^111^In-CP04, t_R_ 9.0 min: ^111^In-CP04, overall radiochemical purity of the preparation >95%.

A sample radiochromatogram is shown in Fig. 2.

### 3.3. Characterisation of non-radioactive impurities

**Fig. 3:**
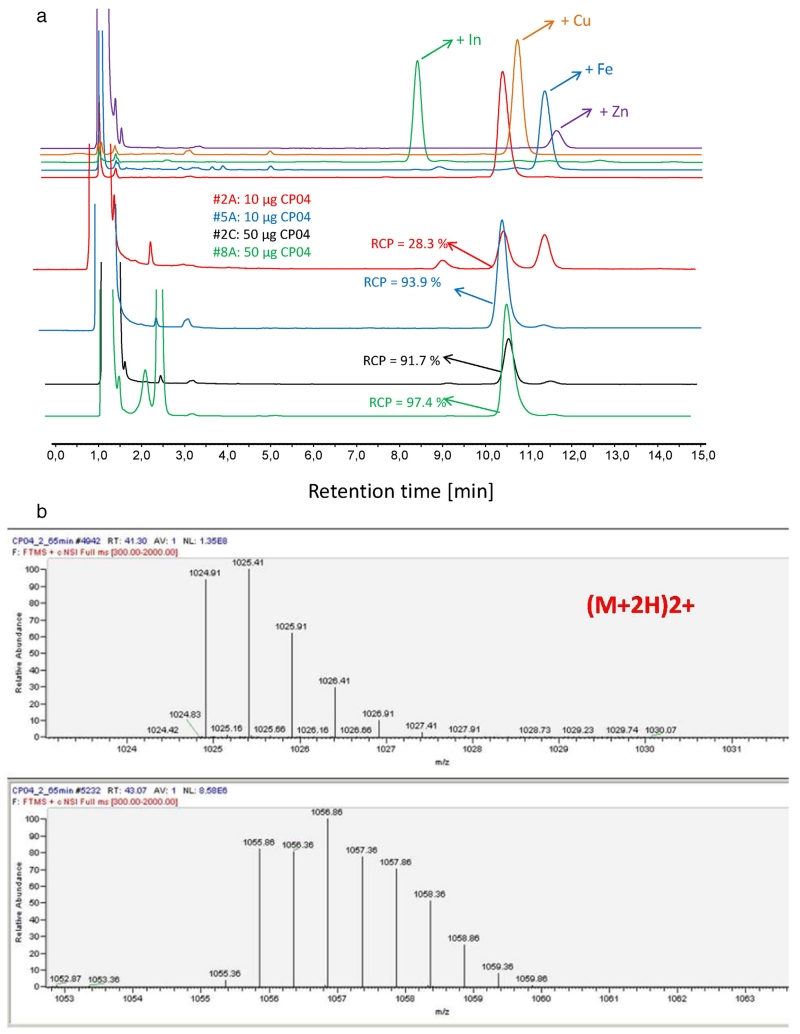
a. Top chromatograms: UV trace (280 nm) during HPLC analysis (system 2) of standards of CP04 (red) and different metal complexes thereof (indium, copper, iron and zinc). Lower chromatograms: UV-trace (280 nm) of kit samples dissolved in metal free water, blue and red traces represent 10 μg kit batches, green and black 50 μg kit batches with added RCP values after radiolabelling of different vials of the same batch (red and black poor, blue and green good radiolabelling, corresponding to the presence of a second peak) b: Identification of corresponding Zn-complex in kit # 2 associated with A, as shown in comparative mass spectra of the main CP04 peak (top, HPLC-t_R_10.5 min) and the Zn-complex (bottom, HPLC-t_R_ 11.5 min) with a mass addition of 62 Da and characteristic isotopic distribution of Zn. (For interpretation of the references to colour in this figure legend, the reader is referred to the web version of this article.)

By dissolving the kits in 1 mL metal-free water and analysing the solution by HPLC (system 2) during the development process additional UV peaks (t_R_ 11.5 min) eluting close to CP04 (t_R_ 10.5 min) were detected in varying amount (Fig. 3a). The higher the ratio of these unknown peaks over CP04 the lower the obtained radiolabelling yields were, especially in the 10 μg formulation.

This phenomenon was attributed to trace-metal contamination during preparation of the kit and interference with radiolabelling due to the very low amount of CP04 used. To test this hypothesis, authentic samples of CP04 complexed with various metals were prepared and their HPLC profile determined. These complexes eluted with different retention times, as shown in Fig. 3a (e.g., “free” CP04 10.5 min, In-CP04 8.5 min, Cu-CP04 10.8 min, Fe-CP04 11.3 min, Zn-CP04 11.5 min). In addition, LC–ESI-MS analysis was performed in one research batch, revealing Zn-CP04 as the main impurity on HPLC (Fig. 3b: mass + 62 as compared to “free” CP04 and characteristic isotope distribution for the Zn-complex).

### 3.4. Kit formulation development

Table 2 summarises the results from all produced kit batches. Only batches 5C, 8, 9 and 10 resulted in sufficiently high RCP after radiolabelling the low dose (10 μg) formulation. Initial batches (1–4) showed high amounts of free ^111^In, whereby labelling of high dose (50 μg) kits resulted in RCP of >90% in two cases (batch 2C and 3C). Additionally, variable levels of oxidised ^111^In-CP04 — between 1.0 and 10.5% — were found. In batch 5C l-methionine and gentisic acid was added and compared with the same formulation without these additives (batch 5A and 5B), showing the amount of oxidised ^111^In-CP04 considerably reduced from >5 to 1.9%. However, the same formulation failed to provide high RCP in subsequent batches (6 and 7). Further-more, after identification of Zn-contamination during the formulation process (most likely introduced during the washing process of 10 mL vials), 2 mL vials were used leading to higher RCP values (>95%) in the final formulation (batches 8, 9 and 10). Given that no significant influence was seen on RCP by varying the amount of ascorbic acid (25–75 mg), the lower amount was chosen for the final formulation. The batches of the final formulation (Table 3) showed very comparable results at both low dose (10 μg) and high dose (50 μg) formulations in 4 subsequent batches (Table 4).

### 3.5. Master batch data

Five GMP, clinical grade kit batches (9A, 9B, 10A, 10B, 11) were tested 3 times for all specifications. Data are summarised in Table 4. All kits were sterile and revealed low levels of endotoxins, appearance dissolution and pH were according to specifications. Residual water was between 2.7 and 3.1%. HPLC analysis revealed intact, non-oxidised or metal bound CP04 within 10% of the stated amount and RCP exceeded 95% in all tests. Overall all batches passed release testing.

### 3.6. Stability studies

Kits with the final kit composition (No. 5, 8 and 9, Table 3) were radiolabelled after different storage times and the results are summarised in Table 5. All samples showed no changes in pH, appearance or CP04 content and RCP values exceeded 95%, except for one sample which was stored at 25 °C for 3 months with a RCP value of 94.8%. No significant trend towards lower RCP values or new radiochemical impurities at longer storage times were detected. Additional parameters, such as appearance before and after dissolution, or pH did not show any undesirable changes within the observation period.

Test results on the stability of the radiolabelled products are shown in Table 6. Up to 4 h after labelling no significant change in RCP was detected and only after 24 h a trend towards lower RCP was seen, although very limited with RCP values of >94%. Additional tests also revealed no decrease in RCP values during storage in the vial or in syringes over a period of 4 h and no indication of adsorption on the wall of syringes or vials (data not shown).

## 4. Discussion

The development of a radiotracer from the research phase into a clinically applicable (radio)pharmaceutical for first-in-human clinical trials is a challenging task (Reilly et al., 2015) and needs to abide to particular guidelines as compared to “conventional” drugs. It should be taken into account that a kit formulation, as presented here, is not used as such in patients, but only serves as a platform for the on-site preparation of the actual radiopharmaceutical. The addition of a radionuclide precursor (^111^In-chloride) solution to a kit following a predefined procedure will yield the final radiopharmaceutical for intravenous injection to patients. Accordingly, the development of such kit formulation is primarily focused on ensuring the successful performance of the kit during radiolabelling, monitored by HPLC analysis of the radiolabelled drug, rather than on testing the kit before radiolabelling, to safeguard the efficacy of the radiopharmaceutical.

In this particular case, a peptide-ligand targeting CCK2 receptors and modified with a chelator to bind a radiometal, here ^111^In, had to be formulated to ensure almost quantitative binding of the radiometal. In general, for radiopharmaceuticals the unlabelled (non-radioactive) precursor should not saturate the target to comply with the so-called tracer principle (Konijnenberg et al., 2014). In the present formulation intended for the use in a first-in-human study, a very low amount of CP04 (the precursor) was chosen to be administered in patients. For safety reasons the study will be initiated starting with only 10 μg CP04 (4.9 nmol), which is the active substance in the formulation (Committee for human medical products (CHMP), 2008). A second higher dose (50 μg) formulation has also been included for use in a subsequent phase of this clinical trial (Decristoforo et al., 2015).

This very small amount of peptide is highly challenging to handle. Although comparable formulations are on the market, e.g., Octreoscan^®^ (Summary of Product characteristics Octreoscan, 2004), very little detail is reported in the literature for radiolabelling of freeze-dried peptide-conjugates with trivalent metals intended for molecular targeting applications. The amount of the DOTA-conjugated peptide is much lower than in comparable formulations of antibodies or small proteins, that have been reported for ^111^In-labelling (Scollard et al., 2011; Reilly et al., 2004). Another difference is that the described kit formulations use DTPA as chelator for ^111^In, whereas in this case DOTA is used. To our knowledge this is the first report on a freeze-dried kit formulation of a DOTA-conjugated peptide. DOTA is known to form stable complexes with a variety of metals, such as copper, zinc or ferric ions, that are frequently present in the environment and easily introduced during the formulation process and interfere with radiolabelling (Breeman et al., 2003). Last but not least, CP04 contains the oxidation-prone Met^11^ residue in the receptor-binding site of the peptide chain, which upon oxidation leads to complete loss of CCK2-receptor affinity and thus total loss of its targeting efficacy (von Guggenberg et al., 2009).

These challenges could be positively addressed step-by-step in the present work, as described in more detail below.

### 4.1. Preformulation

Although the sodium acetate buffer with seleno-dl-methionine showed high RCP, it has limitations for kit formulation development. Seleno-dl-methionine raises toxicity and safety concerns when injected intravenously, whereas an acetate buffer formulation cannot be freeze-dried and may have limitations regarding stability. Based on that, an ascorbic acid formulation (de Blois et al., 2012) was chosen instead for further kit development.

### 4.2. Kit development and impurities

19 development freeze-dried batches were prepared to finally come to a suitable kit formulation. The major parameters were successfully addressed, resulting in a suitable product for clinical application.

A low amount of ascorbic acid of 25 mg was sufficient, given that a higher content did not improve radiopharmaceutical quality but only increased the risk of trace-metal contamination. A detailed investigation on the composition of the radiopharmaceutical precursor in the kit was required to identify the source of trace-metal contaminants. The presence of Zn-CP04 by-product was clearly identified by LC–MS, both by the mass and by the isotopic profile in the MS signal. Although the exact source of Zn contamination was not found, e.g., metal removal in the water used by ion exchange resin did not improve the result, the problem could be eventually resolved by a combination of actions, such as changing of container size from 10 to 2 mL, general use of low metal containing solutions and non-metallic instruments during the washing process. In particular, the careful selection of coated containers ensured no detectable adsorption of peptide on the glass surface, the amount of precursor was within 10% of the predicted value. Test measuring the residual activity after withdrawing the radiolabelled solution into syringes confirmed this (<5% remaining radioactivity).

Addition of methionine and gentisic acid as antioxidants (de Blois et al., 2012) increased RCP up to >95% mainly due to the suppression of Met^11^-oxidation of the CP04 precursor. These anti-oxidants also resulted in excellent stability of both the kit and the radiolabelled product.

The formulation was successfully translated to four subsequent GMP/clinical grade batches of CP04 kits for ^111^In-labelling, passing all predefined quality control tests both related to the kit itself (pH, appearance, sterility, endotoxins, CP04 content), as well as related to the radiolabelling step (RCP).

### 4.3. Stability

The described final formulation (Table 4) showed excellent radiolabelling yield and radiochemical purity after storing the kits up to 1 year in a refrigerator (5 °C) or up to 6 months at 25 °C (Table 5). Therefore, the shelf life has been set to 1 year thus far. However, results from ongoing tests may potentially extend storage times. In view of the fact that no degradation in quality has been registered up to now, it is likely that much longer shelf lives will finally be defined. Moreover, stability of the radiolabelled product in solution is excellent in the current formulation, considering the resistance of the peptide to oxidation, the absence of quality deterioration as long as 4 h after labelling and the only minimal decrease in purity 24 h after labelling.

Overall this data indicates compliance of the described formulation with the requirements for radiopharmaceutical preparations, as outlined in the respective guidelines of the European Medicines Agency (Committee for human medical products (CHMP), 2008). Further experiments to reproduce these findings in a therapeutic setting by switching the radionuclide from ^111^In to the β-emitter ^177^Lu are underway. So far the described formulation has passed the evaluation process by the pharmaceutical (“competent”) authorities and ethical committees in three participating European countries, while three applications are pending.

## 5. Conclusion

A promising drug candidate, a radiopeptide targeting the CCK2 receptor initially intended for a first-in-man diagnostic and dosimetric study, was successfully developed into a kit formulation for radiolabelling with ^111^In, whereas data on preclinical pharmacology, dosimetry and toxicity testing will be reported separately. The described formulation will be used in a first multi-centre clinical trial in MTC patients with the aim to contribute to the improvement of the diagnosis and therapy of metastatic MTC.

## Figures and Tables

**Table 1:** Summary of initial ^111^In-labelling experiments: influence of buffers, incubation times and incubation temperatures on radiochemical purity. (Radiolabelling: 20 μg CP04,50 MBq ^111^In).

Buffer solution	Incubation time [min]	Temperature [°C]	Free ^111^In [%]	Oxidised CP04 [%]	^111^In-labelled CP04[%]
Ascorbic acid buffer (50 mg/0.2 mL H_2_O)	15	75	4.3	1.7	93.6
	30	75	1.3	3.5	94.4
	15	85	11.5	6.6	78.3
	30	85	6.2	8.1	81.7
	15	95	2.2	8.5	84.9
	30	95	2.2	10.2	81.8
Sodium acetate buffer 0.8 M/Se-Met (0.01 mg/μL)	15	75	1.4	1.4	93.8
	30	75	1.3	1.5	93.7
	15	85	0.5	5.5	90.3
	30	85	0.6	5.7	89.4
Sodium acetate buffer (0.8 M)	15	75	0.6	3.8	92.6
	30	75	0.6	5.1	91.2
	15	85	17.7	2.4	79.0
	30	85	17.1	3.5	78.2

**Table 2:** Overview of all CP04 kit batches including production details and main QC-results (Nd = not determined).

		Production details					Quality control results				
Batch no.	CP04 stated content [μg]	Batch size [number of vials]	Ascorbic acid [mg]	Methionine [μg]	Gentisic acid	Vial size [mL]	CP04 content measured [μg] (UV)	Intact CP04 [%] (UV)	RCP [%]	Free ^111^In[%]	Oxidised ^111^In-CP04[%]
1A	10	15	50	0	0	10 mL	7.5	63.6	4.4	95.6	0
1B	10	15	60	0	0	10 mL	6.8	64.2	Nd	Nd	Nd
1C	50	15	75	0	0	10 mL	6.6	64.3	10.3	89.7	0
2A	10	15	50	0	0	10 mL	10.2	54.6	28.3	70.6	1.1
2B	10	15	75	0	0	10 mL	9.4	67.2	67.8	26.8	5.4
2C	50	15	75	0	0	10 mL	52.3	91.8	91.7	3.6	4.5
3A	10	15	25	0	0	10 mL	8.0	70.3	88.2	1.4	10.4
3B	10	15	50	0	0	10 mL	8.0	61.8	81.6	8.2	10.2
3C	50	15	25	0	0	10 mL	41.9	91.7	92.4	0.8	6.8
4	10	20	25	0	0	10 mL	11.3	19.2	55.7	40.9	3.4
5A	10	25	25	0	0	2 mL	12.5	97.0	93.9	0.3	5.8
5B	20	25	25	0	0	2 mL	25.0	97.9	94.4	0.3	5.3
5C	10	25	25	5.2	0.53	2 mL	11.0	90.4	97.7	0.4	1.9
6A	10	50	25	5.2	0.53	10 mL	12.9	52.4	86.1	Nd	Nd
6B	10	50	25	5.2	0.53	10 mL	14.1	56.5	63.2	36.8	0
7A	10	50	25	5.2	0.53	10 mL	12.5	35.8	49.3	Nd	Nd
7B	50	50	25	5.2	0.53	10 mL	59.6	84.4	< 5	Nd	Nd
8A	50	50	25	5.2	0.53	2 mL	71.2	98.2	98.0	0.5	1.5
8B	10	50	25	5.2	0.53	2 mL	10.7	97.00	98.3	0.4	1.3
9A (GMP)	50	100	25	5.2	0.53	2 mL	44.6	95.3	96.1	0.7	3.2
9B (GMP)	10	100	25	5.2	0.53	2 mL	9.3	91.7	96.5	0.7	2.8
10A (GMP)	50	44	25	5.2	0.53	2 mL	44.8	96.5	96.7	0.3	3.0
10B (GMP)	10	44	25	5.2	0.53	2 mL	9.0	92.2	96.4	0.3	3.2

**Table 3:** Final kit composition.

Names of ingredients	Amount	Function	Quality requirements
CP04TFA salt (DOTA–DGlu–DGlu–DGlu–DGlu–DGlu–DGlu– Ala–Tyr–Gly–Trp–Met–Asp–Phe–NH_2_)	10 μg or 50 μg	Active substance	GMP certification
l-Ascorbic acid	25 mg	Excipient antioxidant, stabiliser	Pharmaceutical grade
Gentisic acid	0.53 mg	Excipient antioxidant	In house specification
l-Methionine	5.2 mg	Excipient antioxidant, stabiliser	Pharmaceutical grade
Sodium hydroxide	3.9 mg	Excipient for pH adjustment	Pharmaceutical grade
Nitrogen	q.s.	Protective gas	99.9999 vol%

**Table 4:** Master batch data of GMP produced clinical grade kit batches (all data mean of N = 3).

1.) 10 μg formulation					
Test	Specification	Batch			
		5C/14	8B/14	9B/14	10B/14
Appearance	White freeze-dried solid	Complies	Complies	Complies	Complies
Appearance after dissolution in 1 mL of water for injection	Clear, colourless or slightly yellow solution	Complies	Complies	Complies	Complies
Identification of CP04	From 95 to 105% of retention time of the CP04 standard	Complies	Complies	Complies	Complies
Assay of CP04	8 – 12 μg	11.0 μg	10.7 μg	9.3 μg	9.0 μg
Radiochemical purity (after radiolabelling with 220 MBq of ^111^In)	≥90%	97.7%	98.3%	96.5%	96.4%
	≤5% free ^111^In	0.4%	0.4%	0.7%	0.3%
	≤5% ^111^In-CP04ox	1.9%	1.3%	2.8%	3.3%
pH after dissolution in 1 mL of water for injection	4.0–5.0	4.5	4.5	4.5	4.5
Sterility	Passes sterility test	Passes	Passes	Passes	Passes
Bacterial endotoxins	<20 EU/kit	<1.25 EU/kit	<1.25 EU/kit	<1.25 EU/kit	<0.25 EU/kit

2.) 50 μg formulation				
Test	Specification	Batch		
		8A/14	9A/14	10A/14
Appearance	White freeze-dried solid	Complies	Complies	Complies
Appearance after dissolution in 1 mL of water for injection	Clear, colourless or slightly yellow solution	Complies	Complies	Complies
Identification of CP04	From 95 to 105% of retention time of the CP04 standard	Complies	Complies	Complies
Assay of CP04	40–54 μg	–	44.6 μg	44.8 μg
Radiochemical purity (after radiolabelling with 220 MBq of ^111^In)	≥ 90%	98.0%	96.1%	96.7%
	≤5% free ^111^In	0.5%	0.7%	0.3%
	≤ 5% ^111^In-CP04ox	1.5%	3.2%	3.0%
pH after dissolution in 1 mL of water for injection	4.0–5.0	4.5	4.5	4.5
Sterility	Passes sterility test	Passes	Passes	Passes
Bacterial endotoxins	<20 EU/kit	<1.25 EU/kit	<1.25 EU/kit	<0.25 EU/kit

**Table 5:** Long term and accelerated stability data of different batches of the CP04 kits (Radiochemical purity after radiolabelling with ^111^In (nd = not determined/analysed).

	Time	Storage 5 °C ± 3°C			Storage 25 °C (“accelerated”)	
Batch		5	8	9	8	9
10 μg formulation	Release	97.7	97.6	96.1	97.6	96.1
	1 month	nd	nd	nd	97.1	95.7
	3 months	96.7	96.5	95.7	96.5	94.8
	6 months	97.0	97.8	96.1	95.9	95.3
	9 months	nd	96.3	nd	nd	nd
	12 months	nd	nd	95.9	nd	nd
50 μg formulation	Release	nd	97.4	95.6	97.4	95.6
	1 month	nd	nd	nd	97.1	96.1
	3 months	nd	97.0	96.1	97.5	95.1
	6 months	nd	96.9	96.0	97.1	92.9
	9 months	nd	96.7	nd	nd	nd
	12 months	nd	nd	95.2	nd	nd

**Table 6:** Stability data of ^111^In-CP04 preparations from kits (Radiochemical purity at indicated time points).

Batch no.	CP04 content [μg]	After radiolabelling	2 h post preparation	4 h post preparation	24 h post preparation
5C	10	96.7	97.5	97.1	95.9
8A	50	97.1	97.1	97.0	96.8
8B	10	97.1	97.1	97.1	96.8
9A	50	95.6	95.6	95.6	95.0
9B	10	96.1	95.4	95.4	94.4
